# New transgenic NIS reporter rats for longitudinal tracking of fibrogenesis by high-resolution imaging

**DOI:** 10.1038/s41598-018-32442-x

**Published:** 2018-09-21

**Authors:** Bethany Brunton, Lukkana Suksanpaisan, Hongtao Li, Qian Liu, Yinxian Yu, Alyssa Vrieze, Lianwen Zhang, Nathan Jenks, Huailei Jiang, Timothy R. DeGrado, Chunfeng Zhao, Stephen J. Russell, Kah-Whye Peng

**Affiliations:** 1 Department of Molecular Medicine, Mayo Clinic, Rochester, MN USA; 2 Imanis Life Sciences, Rochester, MN USA; 3 Department of Orthopedic Surgery, Mayo Clinic, Rochester, MN USA; 4 Department of Radiology, Mayo Clinic, Rochester, MN USA

## Abstract

Fibrogenesis is the underlying mechanism of wound healing and repair. Animal models that enable longitudinal monitoring of fibrogenesis are needed to improve traditional tissue analysis post-mortem. Here, we generated transgenic reporter rats expressing the sodium iodide symporter (NIS) driven by the rat collagen type-1 alpha-1 (Col1α1) promoter and demonstrated that fibrogenesis can be visualized over time using SPECT or PET imaging following activation of NIS expression by rotator cuff (RC) injury. Radiotracer uptake was first detected in and around the injury site day 3 following surgery, increasing through day 7–14, and declining by day 21, revealing for the first time, the kinetics of Col1α1 promoter activity *in situ*. Differences in the intensity and duration of NIS expression/collagen promoter activation between individual RC injured Col1α1-hNIS rats were evident. Dexamethasone treatment delayed time to peak NIS signals, showing that modulation of fibrogenesis by a steroid can be imaged with exquisite sensitivity and resolution in living animals. NIS reporter rats would facilitate studies in physiological wound repair and pathological processes such as fibrosis and the development of anti-fibrotic drugs.

## Introduction

Type I collagen is abundantly expressed in various normal organs including bones, skin and teeth^1^. It is also a major component of fibrotic scar tissue formed during wound repair in a process known as fibrogenesis^1^. Inadequate fibrogenesis leads to poor healing and delayed tissue regeneration. In contrast, excessive fibrogenesis causes organ fibrosis, which is a common cause of liver, lung, heart or kidney failure^2^. Drugs or therapies that could stimulate connective tissue (e.g. type I collagen) synthesis may accelerate surgical/traumatic wound healing and inhibition of its synthesis may prevent or reverse organ failure. However, despite its biomedical importance, fibrogenesis remains a poorly accessible drug target due to the current difficulties in studying and visualizing connective tissues *in vivo*.

Rodents are the most common mammals used as preclinical animal models of disease to study basic cellular biology, disease modeling and for the development and testing of novel therapeutics^3,4^. A typical preclinical *in vivo* study involves harvesting organs from numerous animals at various time points for histological and biochemical analysis of the tissues postmortem. This is labor intensive, expensive, and importantly, does not allow serial longitudinal tracking of biological processes in the same animal over time. Due to these limitations, noninvasive imaging technologies are highly attractive, and is line in with supporting the 3Rs in animal research, significantly reducing the number of animals used in research^5^.

The recent development and improvement of noninvasive imaging using the sodium iodide symporter (NIS) reporter gene has provided an excellent technology for longitudinal tracking of pharmacokinetics and the fate of gene, cell and virus therapies in living animals^6–8^. NIS is the thyroidal sodium iodide symporter, which concentrates radioiodine and related radioactive anions (such as Tc-99 m pertechnetate and ^18^F-tetrafluoroborate (^18^F-TFB)) in NIS-expressing cells^6,8–10^. NIS is used clinically for diagnosis of thyroid disorders through radioiodine gamma camera imaging^9,11^. The NIS gene has also been introduced into non-thyroid cells (eg. fibroblasts and mesenchymal stem cells)^12,13^ and expression has been detected by radioiodine imaging^14,15^. Importantly, because gamma rays are able to penetrate deep tissues, NIS has been used successfully as a reporter gene to monitor the transplantation and regeneration of diseased liver by healthy NIS-expressing hepatocytes from small (mice) to large animals (pigs)^16,17^. Single-photon emission computed tomography (SPECT) and positron emission tomography (PET) scanners are used to image and quantify NIS-mediated radiotracer uptake. PET and SPECT imaging modalities are superior to optical imaging modalities (fluorescence or bioluminescence) for allowing quantitative imaging of body parts deep within the body. Since NIS is a self-protein (i.e. non-immunogenic) and its orthologues sequences are known for various species^18–20^, it is suitable for long term *in vivo* tracking of cells overtime in different animal species. Furthermore, the NIS reporter gene can serve to noninvasively track not only normal physiological repair *in vivo* but also pathological processes such as fibrosis, inflammation, oncogenesis and metastasis.

To date, connective tissue synthesis during both normal and pathological conditions and its modulation by drug therapy have been difficult to study in living animals. Previous studies have created transgenic animals expressing various collagen promoter-driven reporter genes (CAT, β-gal, GFP, and luciferase)^21–25^ but none of these models could be used for high resolution live monitoring of collagen synthesis. Recently, noninvasive imaging using transgenic collagen 1 alpha 1 promoter (Col1α1)-driven-luciferase mice was attempted^25^, however, bioluminescence images are inherently limited by depth of penetration of optical photons suggesting the need for improved noninvasive methods. Furthermore, rats are commonly used for musculoskeletal research and highly valued as a model in many preclinical studies due to their larger size, facilitating surgical procedures and more frequent blood sampling.

Here, we have developed transgenic collagen promoter (Col1α1)-hNIS reporter rats for noninvasive longitudinal in-life monitoring by nuclear imaging (SPECT or PET/computed tomography (CT)) of collagen promoter activation following tissue injury. Specifically, we utilized the rat rotator cuff (RC) injury model to demonstrate collagen promoter activation and fibrogenesis following injury. SPECT or PET/CT detection of NIS expression qualitatively correlated with collagen expression following injury in the transgenic rats. Treatment with dexamethasone, a steroid known to alter the process of collagen synthesis, delayed collagen promoter activation in the transgenic rats. These transgenic rodents demonstrate for the first time the utility of NIS as a reporter gene to monitor collagen promoter activation following injury for in-depth analysis of the process of fibrogenesis in living animals.

## Results

### Creation of Col1α1-hNIS transgenic rats

**Figure 1:**
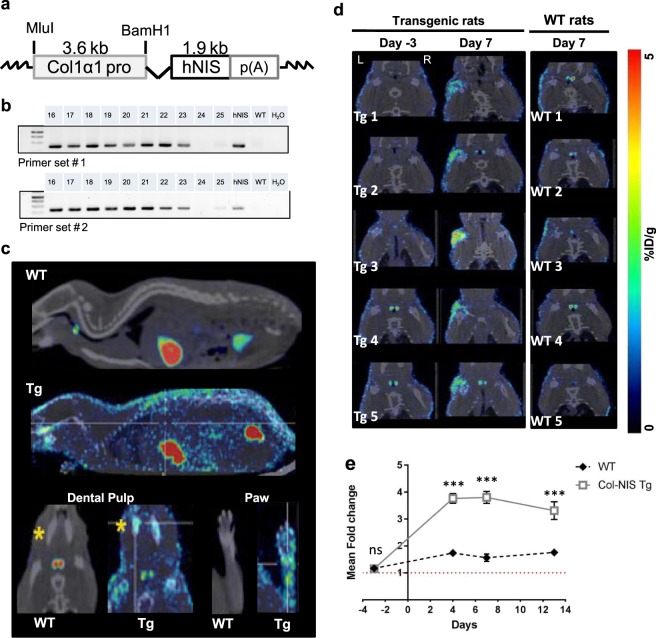
Characterization of Col1α1-hNIS transgenic rats. (**a**) Schematic of the collagen type 1 alpha 1 (Col1α1) promoter driven human NIS (hNIS) cDNA inserted into an expression plasmid for creation of the Col1α1-hNIS transgenic rats. (**b**) PCR genotyping identified Col1α1-hNIS transgenic HSD rats. Genomic DNA from tail snips was screened for the hNIS transgene with two independent primer sets. The correct genotype was identified by the presence of a 314 (primer set #1) or 324 bp (primer set #2) PCR product. Lane designations represent single transgenic rat along with hNIS plasmid DNA (positive control), wild type (WT) rat DNA (negative control) and water control. (**c**) Representative SPECT/CT images demonstrate NIS expression in the skin, dental pulp (mandibles) and joints (paws) of Col1α1-hNIS transgenic rats but not in non-transgenic rats. (**d**) Rotator cuff injury increases NIS-mediated Tc-99 m pertechnetate uptake at the injury site in Col1α1-hNIS transgenic rats. Only the left shoulders of Col1α1-hNIS transgenic rats have increased Tc-99 m pertechnetate signal at 7 days following rotator cuff injury, even though the left shoulder of the non-transgenic WT rats was also injured. Right shoulder of each rat served as the uninjured negative control for radiotracer uptake. (**e**) Quantification of NIS-mediated Tc-99 m pertechnetate uptake overtime in the left shoulder of Col1α1-hNIS rats following rotator cuff injury. Data represents mean fold increase of the Tc-99 m pertechnetate signal on the left, injured shoulder over the right, uninjured control shoulder of each animal. N = 5 for each rat genotype. Significance was determined by two sample t-test analysis at each time point, ***p < 0.001, ns = not significantly different.

Here, we created a novel transgenic reporter rat to evaluate the feasibility of using NIS as a reporter gene to monitor the pathological process of fibrogenesis. The human NIS (hNIS) cDNA under the control of the type I collagen alpha 1 promoter (Col1α1) was subcloned into an Adeno-associated virus (AAV) expression vector (Fig. 1a). To generate transgenic rats, the DNA construct was injected into Harlan Sprague Dawley (HSD) zygotes and transferred into pseudopregnant female recipients primed using hormonal injections to receive the embryos as previously described^26^. Genotype was verified by PCR amplification of hNIS, not rat NIS, using two different hNIS primer sets (Fig. 1b and Supplementary Fig. 1). Additionally, the rats were screened by SPECT/CT imaging to detect radiotracer Tc-99 m pertechnetate uptake. Physiologic uptake of Tc-99 m pertechnetate is seen in tissues that normally express NIS, such as the thyroid and gastric mucosa (Fig. 1c). In addition, high Tc-99 m pertechnetate uptake was also detected in tissues expected to have high collagen expression including the skin, dental pulp (mandibles), and joints (paws) of the Col1α1-hNIS transgenic rats (Fig. 1c). In contrast, little to no Tc-99 m pertechnetate uptake was observed in the skin, mandibles or paws of non-transgenic HSD rats confirming that hNIS expression was responsible for the increased radiotracer uptake in the transgenic animals.

### Tracking fibrogenesis by noninvasive imaging

The Col1α1-hNIS transgenic rats were used to evaluate the feasibility of using NIS reporter gene imaging to monitor repair and healing following RC injury in living animals as a model of fibrogenesis. Non-transgenic and Col1α1-hNIS transgenic rats underwent surgery induced RC injury on their left shoulders. The right, uninjured shoulder served as a negative control. Animals were monitored through serial SPECT/CT imaging over 14 days. Prior to imaging, rats received 1 mCi Tc-99 m pertechnetate given intraperitoneally 1 hour before being scanned. Negligible or no Tc-99 m pertechnetate uptake was observed by SPECT imaging in the left shoulder joints of non-transgenic and transgenic rats at baseline three days prior to injury (Fig. 1d). At 7 days following RC injury, significant NIS-mediated Tc-99 m pertechnetate uptake was observed encircling and surrounding the left injured shoulder of the transgenic rats compared to the non-transgenic rats (Fig. 1d and Supplementary Video 1). Quantitative analysis of the SPECT data indicates a significant increase (3 to 5 fold) in NIS mediated Tc-99 m uptake in the injured shoulder over control shoulder in the transgenic animals compared to wildtype (WT) animals. Peak NIS signals occurred at day 7 following injury (Fig. 1e).

**Figure 2:**
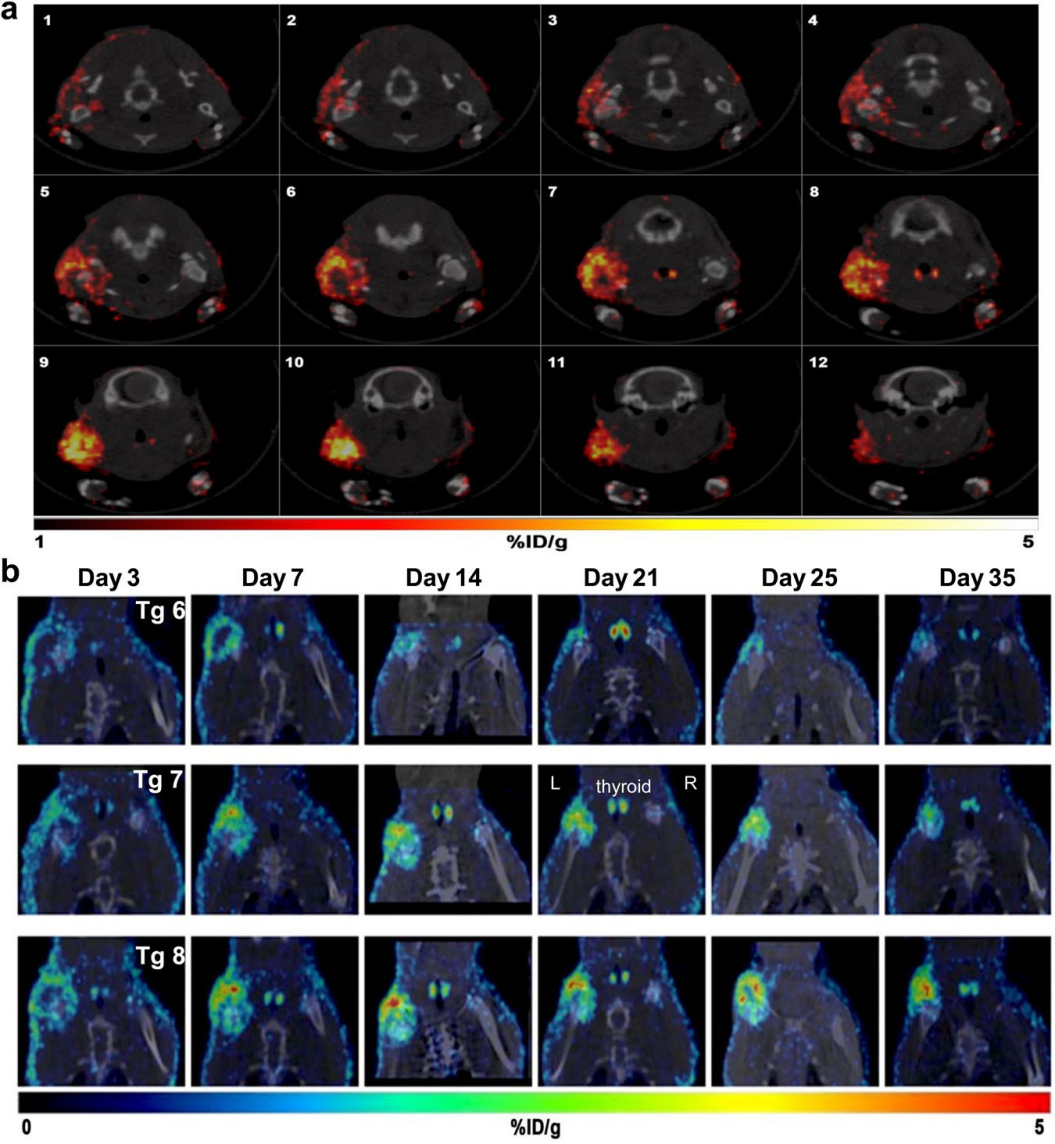
Longitudinal monitoring of activation of the Col1α1 promoter in transgenic rats after rotator cuff injury. (**a**) Transverse serial SPECT/CT fusion images from a Col1α1 transgenic rat with RC injury (left shoulder) allows in depth analysis of areas of high Col1α1 activity as indicated by high tracer uptake. (**b**) Longitudinal SPECT/CT images from three transgenic rats during acute and recovery phases of RC injury on the left shoulder. Right shoulder was not injured and serves as the negative control.

Because SPECT data is tomographic, we are able to easily obtain spatial information at any time point without having to euthanize the animal. Figure 2a shows serial (every 3 sections) transverse SPECT/CT fusion images, starting at the point where the first NIS signal was evident at the injured shoulder, to the cessation of the NIS signal. It is evident that the skin has intense NIS activity where there is strong activation of the Col1α1 promoter from the surgery. Subsequent images show defined areas of NIS signal further into the RC cut site (Fig. 2a). From the SPECT imaging data of the 3 representative transgenic rats shown in Fig. 2b, it is evident that extent of injury and subsequent host response to the RC injury is variable. In some animals (e.g. Tg 8) NIS activity was higher and remained high to day 35 (last imaging time point) and could be due to more extensive injury in that specific animal. In some animals (e.g. Tg 6), the NIS signals have decreased significantly by day 35 (Fig. 2b).

**Figure 3:**
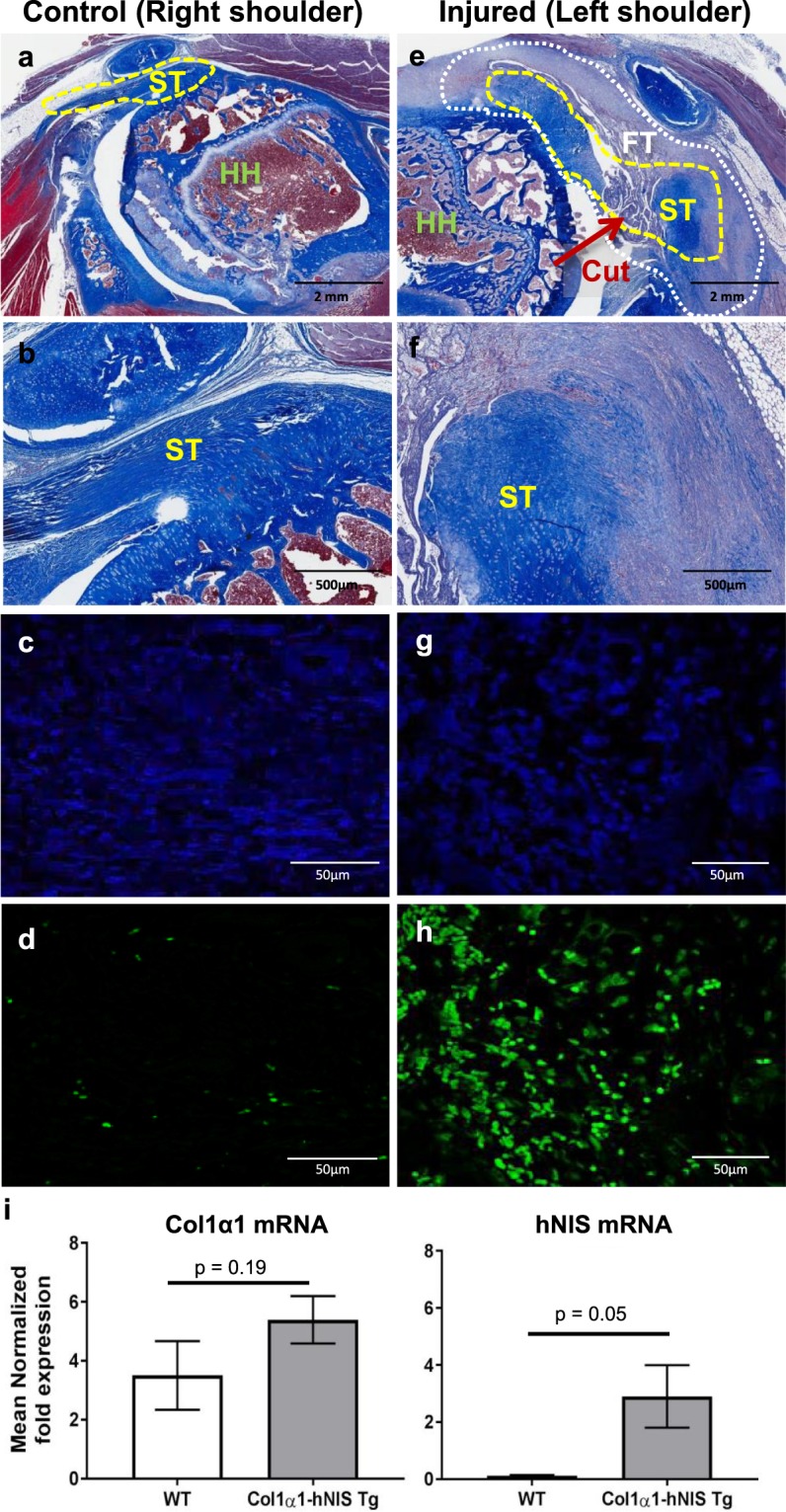
Increased fibrotic tissue formation and hNIS positive cells in rotator cuff injury tissue from a Col1α1-hNIS transgenic rat. Shoulder tissue was collected 8 days following RC injury for histological analysis from a representative Col1α1-hNIS transgenic rat. (**a**,**e**) Masson Trichrome stained images of the humeral head area of the shoulder tissue (20× magnification). HH, humeral head; ST, supraspinatus tendon; FT, Fibrotic tissue outlined with white dotted line. The yellow dotted line designates the supraspinatus tendon (ST) and the red arrow demonstrates the location where the tissue was cut to induce supraspinatus tendon injury in the left shoulder of the Col1α1-hNIS transgenic rat. (**b**,**f**) 100× magnification of the ST labeled region in 20x images (**a** and **e**). (**c**,**d**,**g**,**h**) are confocal images (400X magnification) following immunofluorescent staining of the control and injured shoulder tissue paraffin embedded rotator cuff tissue sections with Hoechst stain for nuclei (blue- **c** and **g**) or an affinity purified polyclonal anti-hNIS antibody (green- **d** and **h**). (**i**) Quantitative RT-PCR analysis for type 1 alpha 1 collagen and hNIS mRNA showed increase in hNIS mRNA in injured shoulder compared to control shoulder of transgenic rats (n = 5) compared to WT rats (n = 3). P = 0.05 as determined by Student’s t-test analysis.

Tissues from the injured and non-injured shoulders of five different Col1α1-hNIS transgenic rats were harvested for immunohistochemical staining to confirm activation of NIS protein expression of the NIS imaging positive shoulders as well as tissue injury by Masson trichrome staining for collagen. The tissue section that best demonstrate the humeral glenoid joint with the entire rotator cuff tendons from both the injured and non-injured shoulder is shown in Fig. 3. The Masson trichrome stained uninjured shoulder demonstrated normal cellular morphology and anatomic structure that included the humeral head (HH), non-injured supraspinatus tendon (ST), and surrounding cartilage, bone and muscle (Fig. 3a,b and Supplementary Table 1). In contrast, the injured shoulder of the rat demonstrated retraction of the supraspinatus tendon from the insertion site, where the injury occurred. In this region there was noticeable pathology including a substantial increase in inflammatory cell infiltration and accumulation of fibrotic scar tissue (Fig. 3e,f and Supplementary Table 1). Immunofluorescence staining also confirmed a significant increase in the number of NIS positive cells in the tendon region of the injured shoulders but not in uninjured control shoulders (Fig. 3c,d,g,h and Supplementary Fig. 2). Quantitative RT-PCR for Col1α1 and hNIS mRNA was also performed. There is a 4–5 fold increase in type 1 alpha 1collagen mRNA in the injured shoulders of both WT and Col1α1-hNIS transgenic rats over the uninjured shoulder of the respective animals (Fig. 3i). As expected, there was no significant difference increase of Col1α1 mRNA between these two groups. In contrast, there is significant induction of hNIS mRNA in the injured shoulders of transgenic rats compared to WT rats (Fig. 3i).

### Noninvasive imaging can detect the modulation of fibrogenesis by drug treatment

**Figure 4:**
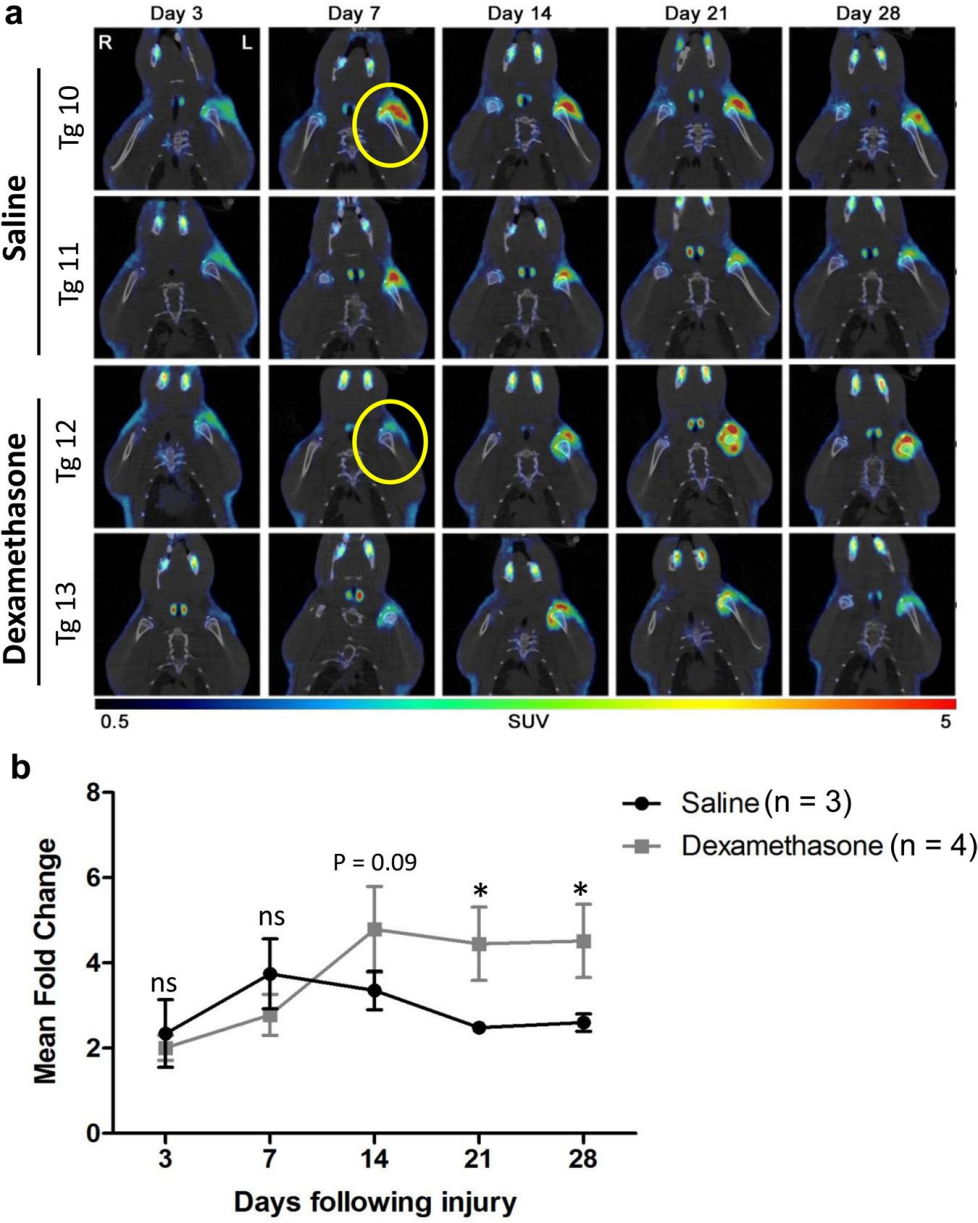
Daily dexamethasone treatment delays collagen promoter activation and NIS signal in Col1α1-hNIS transgenic rats following rotator cuff injury. (**a**) Transverse PET/CT fusion images overtime following injury showing activation of the Col1α1 promoter and positive NIS signals by uptake of ^18^F-TFB in the left shoulder of control (saline treated) and daily dexamethasone (2 mg/kg) treated Col1α1-hNIS transgenic rats. (**b**) Quantification of NIS-mediated ^18^F-TFB uptake overtime in the left shoulder of Col1α1-hNIS rats following rotator cuff injury with and without dexamethasone treatment. (n = 3–4 for each treatment group). Significance was determined by two sample t-test analysis at each time point, *p < 0.05, ns = not significantly different.

Previous studies have demonstrated that the corticosteroid dexamethasone, an immune suppressive drug, is able to significantly reduce inflammation at early time points following tissue injury^27,28^. This reduced inflammation alters tissue organization, fibroblast proliferation, and collagen synthesis during the process of wound healing^27–29^. Using a dexamethasone regimen, we confirmed that the dosage was sufficient to cause decrease in total white blood cell counts of treated rats (Supplementary Fig. 3). Therefore, we subsequently used this steroid to demonstrate that activation of the Col1α1 promoter can be modulated by drug therapy following RC injury. The Col1α1-hNIS transgenic rats received daily subcutaneous treatment with saline or 2 mg/kg of dexamethasone in the left shoulder starting two days prior to and continued daily until 28 days following RC injury surgery. Dexamethasone treatment caused a delay in peak Col1α1 promoter activation with the greatest NIS-mediated ^18^F-TFB uptake by PET/CT being observed 14 days following RC injury (Fig. 4). This is in contrast to the peak promoter activity and isotope uptake being observed at day 7 in the saline treated Col1α1-hNIS transgenic rats (Fig. 4).

## Discussion

In this study, we used newly generated Col1α1-hNIS gene reporter transgenic rats and NIS/radiotracer imaging to demonstrate that the cellular process of repair and fibrogenesis after tissue injury can be monitored noninvasively. SPECT/CT and PET/CT imaging established the feasibility of using the NIS reporter gene under control of a collagen promoter to track activation of cells in tissue repair and regeneration *in vivo* in the same animal over time following injury. Examples of cells that respond acutely to tissue injury as part of the wound healing include fibroblasts, mesenchymal progenitor cells and macrophages^30^. Our SPECT analysis used the standard Tc-99 m pertechnetate tracer that is readily available from the nuclear pharmacy of most hospitals as it is used routinely for thyroid imaging. A standard multiple pinhole SPECT rat collimator, which has a spatial resolution of 0.85 mm, was used to obtain exquisite imaging data on cellular response around the injured shoulder in the RC injury model. Resolution can be improved further using high-resolution collimators (e.g. 0.25 mm) and at least 10-fold higher imaging sensitivity could be achieved using the PET tracer of NIS, ^18^F-TFB, as demonstrated by our dexamethasone experiments^31^. Additionally, we were able to correlate our NIS/radiotracer imaging results with both histological and pathological analyses in the RC model. Previous ‘traditional’ tissue repair *in vivo* studies required many animals that had tissue harvested postmortem at different time points and assessed ‘snapshots’ of the biological processes. In contrast, these hNIS transgenic animals in conjunction with radioisotope imaging provide a way to reduce and refine preclinical animal studies that involve collagen synthesis. Furthermore, we validated the utility of these NIS reporter rats as a valuable research tool whereby immune modulatory or anti-fibrotic drugs could be used to test the safety and efficacy in physiological or pathological wound repair and healing processes.

In contrast to previous studies in Col1α1-reporter animals, we are able to monitor longitudinally the activation of the Col1α1 promoter in cells in response to surgically induced tissue injury in living rats. Beta-galactosidase, CAT, GFP and luciferase reporter genes require euthanasia of the animals at defined time points and harvest of numerous organs for time consuming sectioning, staining and enzymatic or microscopic examination of the tissues^21–24^. Noninvasive *in vivo* imaging of Col1α1-luciferase mice using bioluminescent imaging to monitor bone remodeling and tissue formation has been reported^25^. However, the authors observed very low bioluminescent luciferase signal in adult mice^25^. Since it was not possible to perform *in vivo* bioluminescent imaging in Col1α1-luciferase reporter rats, tissues were homogenized and analyzed *ex vivo* using a luciferase enzymatic assay^32^. In contrast to other studies that have developed integrin and collagen specific molecular imaging probes for detection of fibrosis or fibrogenesis^33–35^, this study uses molecular imaging to monitor activation of the collagen promoter following tissue injury. These integrin and collagen specific imaging probes could be used in combination with the NIS reporter rats to facilitate comprehensive study of fibrogenesis.

We are optimistic that the creation and utilization of such transgenic rodents that express hNIS under different promoters to track biological processes, such as fibrogenesis, or diseases noninvasively has the potential to be a valuable research tool. Additionally, these hNIS reporter rodents could be employed to test the efficacy of novel drugs, new surgical methods or therapies to treat a variety of diseases. These preclinical studies would be able to be performed with fewer animals and utilize less invasive methods but still provide an important understanding of biological processes and diseases *in vivo* that current technologies strive to obtain. Importantly, we begin to appreciate and easily visualize the variability in surgical induced injury, as well as responses of individual animals in the inflammatory and wound healing responses, showcasing the value of noninvasive imaging where superior data could be obtained and providing individualized datasets unique to each test subject.

## Methods

### Creation of transgenic animals

All animal care and experimental procedures were performed in accordance with the relevant guidelines and regulations approved by the Institutional Animal Care and Use Committee of the Mayo Foundation. For generation of the Col1α1-hNIS rodents, the human NIS cDNA (1.9 kb) was subcloned into an AAV expression plasmid downstream of a 3.6 kb fragment of the rat type I collagen alpha 1 promoter. The vectors were injected into rat zygotes and transferred into pseudopregnant female recipients that were treated with luteinizing hormone releasing hormone angonist (LHRHa) as previously described^26^. The transgenic animal core at the University of Michigan utilized Harlan Sprague Dawley (HSD) rats to create the Col1α1-hNIS transgenic rats. The rodent founder lines were screened for the correct transgenic genotype with primers specific for human NIS, not rat NIS. Invitrogen Taq DNA polymerase kit (Cat. 18038–042) was used according to manufacturer’s instructions to amplify hNIS from 50 ng of rodent genomic DNA. The PCR program utilized was: 3 min at 94 °C, 35 cycles of 45 sec. 94 °C, 30 sec. 55 °C, 90 sec. 72 °C and 10 min at 72 °C. The hNIS specific primers were as follows: Set 1 – Forward 5′ GCTCTCCTCCCTGCTAACGACTC 3′ and Reverse 5′ AGACGATCCTCATTGGTGGGCAG 3′; Set 2 – Forward 5′ TGCGTGGCTCTCTCAGTCAAC 3′ and Reverse 5′ AGTTCTTCAGGACCCTTGACC 3′.

### Surgical rotator cuff injury model

Unilateral rotator cuff injury was induced as previously described^36^. Briefly, each rat underwent general anesthesia with 2% isoflurane and oxygen. Surgery was performed on the left shoulder of the animal and the right shoulder served as the non-surgical (injury) control. Injury was created by sharply transecting and resecting 2 mm supraspinatus tendon at its insertion site without repair. The incision was closed with 5–0 Vicryl sutures. Following surgery, the rats were allowed -free cage activities without immobilization. In the dexamethasone treatment studies, rats received saline or 2 mg/kg of dexamethasone sodium phosphate (Fresenius Kabi USA, LLC, Lake Zurich, IL) by subcutaneous injection near the injury site starting 2 days prior to surgery and given daily until completion of the study.

### SPECT/CT and PET/CT Imaging System

Imaging was performed as previously described using in the Mayo Clinic Small Animal Imaging Core using a U-SPECT-II/CT scanner (MILabs, Utrecht, The Netherlands)^7^ or Inveon Multiple Modality PET/CT scanner (Siemens Medical Solutions, Knoxville, TN). ^18^F-TFB for PET/CT imaging was produced by Dr. Timothy DeGrado’s laboratory as previously described^31^. Forty-five minutes to one hour prior to imaging, 0.5–1 mCi of ^99m^Tc pertechnetate was delivered via intraperitoneal injection to rats for SPECT and 0.75 mCi of ^18^F-TFB was delivered via intravenous injection to the rats for PET imaging.

The SPECT module is described in^37^. Micro-CT image acquisition was performed in 4 minutes for normal resolution (169-µm square voxels, 640 slices) at 0.5 mA and 60 kV. Image acquisition time was approximately 20 minutes for SPECT (24 projections at 50 seconds per bed position) with the UHR-RM high resolution total body focused multi-pinhole cylindrical collimator with 75 1.0 mm pinholes, reconstructed resolution <0.85 mm, sensitivity >700 cps/MBq. All pinholes focus on a single volume in the center of the tube and by using an XYZ stage large volumes up to the entire animal can be scanned at uniform resolution^38^. Coregistration of the SPECT and CT images was performed by applying pre-calibrated spatial transformation to the SPECT images to match with the CT images.

For MicroPET/CT imaging, CT image acquisition was performed in 5 minutes with 360 degree rotation and 180 projections at 500 uA, 80 keV and 200 ms exposure. Effective pixel size was 94.59 with transaxial Field of view (FOV) of 96.86 mm and Axial FOV at 90.81 mm. PET Image acquisition began approximately 45 minutes following isotope injection with total acquisition time of 20 minutes. The FOV for PET centered on the shoulder center of the rats and the histogram parameters were set to 1 frame.

### Image Reconstruction and Data Processing

SPECT reconstruction was performed using a POSEM (pixel-based ordered subset expectation maximization) algorithm^39^ with six iterations using 16 subsets as previously described^7^. Briefly, CT data were reconstructed using a Feldkamp cone beam algorithm (NRecon v1.6.3, Skyscan). After reconstruction, SPECT images were automatically registered to the CT images according to the pre-calibrated transformation, and re-sampled to the CT voxel size.

For MicroPET/CT, the CT data were reconstructed using a Feldkamp cone beam algorithm (NRecon v1.6.3, Skyscan) with downsample setting of 2, sight noise reduction and Shepp-Logan filter. PET reconstruction parameters utilized OSEM2D.

Co-registered of SPECT/CT and PET/CT images were further rendered and visualized using the PMOD software (PMOD Technologies, Zurich, Switzerland). A 3D-Guassian filter (0.8 mm FWHM) was applied to suppress noise, and LUTs were adjusted for good visual contrast. Reconstructed images were visualized as both orthogonal slices and maximum intensity projections. Maximal intensity projection videos and three-dimensional rendering of regions of interests were performed on the PMOD software

### Histology and Immunofluorescence

Tissue samples were fixed in 10% neutral buffered formalin, paraffin embedded and sectioned. Sections were stained with hematoxylin-eosin for tissue structure and morphology along with serial sections stained with Masson Trichrome to detect collagen using standard methods. After staining, whole shoulder tissue sections were imaged with the Aperio ScanScope AT Turbo Digital Pathology scanner (Leica Biosystems). Tissue sections were qualitatively assessed by an American College of Veterinary Pathologist board-certified veterinary pathologist, Dr. Ronald Marler. A blinded histopathology report was based on standard pathology descriptions and terminology. For immunofluorescence staining, an affinity purified polyclonal rabbit antibody against human (KELE) NIS (REA010; Imanis Life Sciences) was used, coupled with Alexa Fluor 488-conjugated goat anti-rabbit secondary antibody (Invitrogen, A11008; ThermoFisher Scientific). The nuclei were stained with Hoechst stain (Molecular Probes, H3570). Stained tissue was imaged using a Zeiss LSM 780 confocal microscope (Carl Zeiss Microscopy, LLC).

### Quantitative RT-PCR

Total RNAs were extracted using the RNeasy Plus Universal Kit (Qiagen) to manufacturer’s instructions. To quantify human NIS (hNIS) and collagen type 1 alpha 1 (Col1α1) expression, the qRT-PCR analysis was performed using LightCycler® 480 RNA Master Hydrolysis Probes Kit (Roche diagnostics), with primers and probes as follows: hNIS probe 56-FAM/CTGTCCACC/ZEN/GGAATTATCTGCACCTT/3IABkFQ, hNIS primer 1 5′ AAGCCACTTAGCATCACCA 3′, hNIS primer 2 5′ CTCATCCTGAACCAAGTGACC 3′, Col1α1 probe HEX/CCGGAGGTC/ZEN/CACAAAGCTGAACA/3IABkFQ, Col1α1 primer 1 5′ CATTGTGTATGCAGCTGACTTC and Col1α1 primer 2 5′ CGCAAAGAGTCTACATGTCTAGG. Rat GAPDH gene expression was used as the endogenous control. All PCR reactions were performed using a Light cycler 480 (Roche diagnostics). The results were calculated using the ΔΔCT method (ΔΔCT = (Rotator cuff injured (RC) Ct NIS or Col1α1 - RC Ct GAPDH) - (Normal injured shoulder (N) Ct NIS or Col1α1 - N Ct GAPDH).

### Statistics

T test statistical analyses were performed using JMP Pro version 13 software (SAS Institute, Cary, NC, USA) to compare samples at each time point tested. Results are shown as means ± standard error of the means (s.e.m.). All data points and animals were reported in results and statistical analyses.

## Electronic supplementary material

Supplementary Information

Supplementary Video 1
