# Supplementary material for: New transgenic NIS reporter rats for longitudinal tracking of fibrogenesis by high-resolution imaging
Source: Sci Rep. 2018 Sep 21;8:14209. doi: 10.1038/s41598-018-32442-x (PMC6155090; doi:10.1038/s41598-018-32442-x)
Supplement: Supplementary file 1 — Supplementary Information [file 41598_2018_32442_MOESM1_ESM.pdf]

New transgenic NIS reporter rats for longitudinal tracking of fibrogenesis by high-resolution imaging

Bethany Brunton[1+], Lukkana Suksanpaisan[2+], Hongtao Li[2], Qian Liu[3], Yinxian Yu[3], Alyssa Vrieze[3], Lianwen Zhang[1], Nathan Jenks[1], Huailei Jiang[4], Timothy R. DeGrado[4], Chunfeng Zhao[3], Stephen J. Russell[1], Kah-Whye Peng[1*]

Supplementary Information

Primer set #1

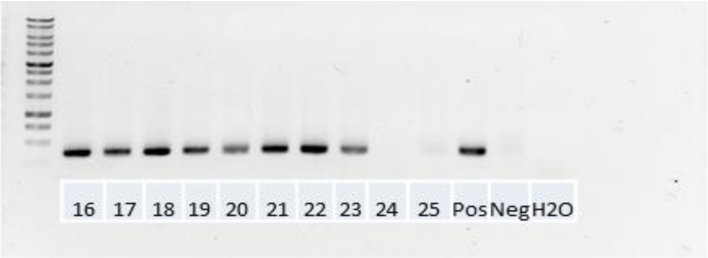

Primer set #2

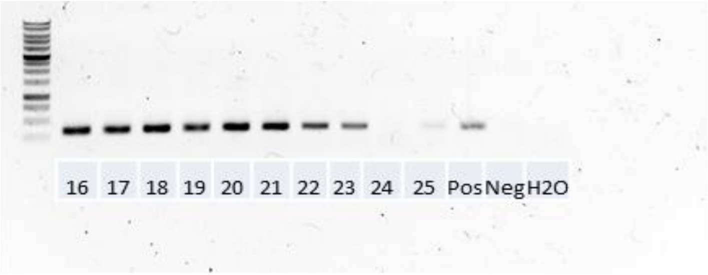

Supplementary Figure 1. Full gel images from PCR genotyping displayed in Figure 1b. Lane designations represent single transgenic rat along with Pos (hNIS plasmid DNA [positive control]), Neg (wild type rat DNA [negative control]) and H2O (water control).

Video attached separately

Supplementary Video 1. Video demonstrating high resolution SPECT/CT imaging of Tc-99m pertechnetate uptake/hNIS signal in a Col1α1-hNIS transgenic rat 7 days following rotator cuff injury of the left shoulder.

Supplementary Table 1. Histological analysis and pathology review of shoulder tissue from Col1a1-hNIS transgenic rats following rotator cuff injury

HH = Humeral Head; Red arrow = RC injury cut site; Dotted white line = Area of fibrotic tissue.

	**Control (Right Shoulder)**		**Injury (Left Shoulder)**	
**Rat ID**	**Masson Trichrome Image**	**Pathology Review**	**Masson Trichrome Image**	**Pathology Review**
Tg 17	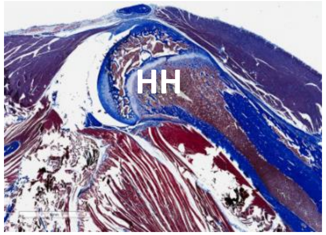	No significant pathology	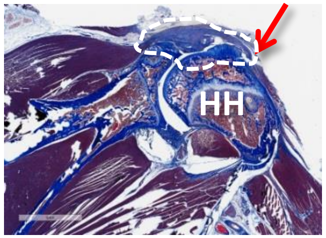	Inflammation, chronic, moderate. Noticeable fibrosis. Inflammation, granulomatous, multifocal, mild. Foreign bodies embedded in the fibrosis and one foreign body with granulomatous inflammation noted in the bone marrow cavity
Tg 18	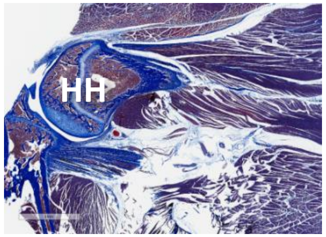	No significant pathology	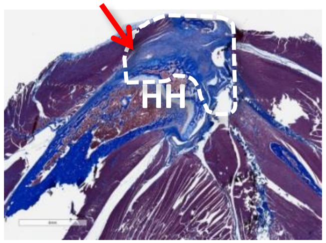	Inflammation, chronic, marked. Considerable fibrosis. Inflammation, granulomatous, multifocal, mild. Foreign bodies embedded in the fibrosis and one foreign body with granulomatous inflammation noted in the bone marrow cavity
Tg 9 **(Detailed depiction in Figure 3)**	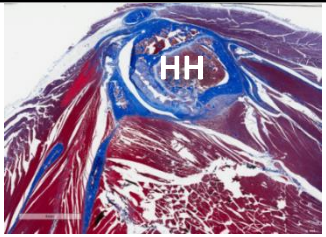	No significant pathology	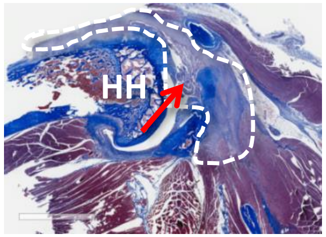	Notable area of periosteal fibrosis adjacent to the joint. Within the primarily fibrous connective tissue proliferation are areas of necrosis and significant inflammatory cell infiltrate
Tg 19	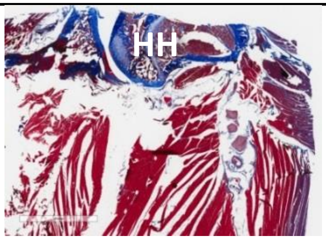	No significant pathology	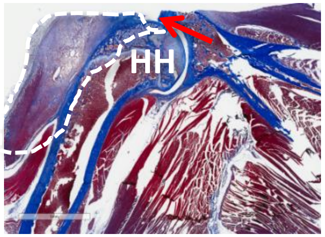	Notable periarticular/articular fibrosis with inflammatory cell infiltrate and necrosis
Tg 20	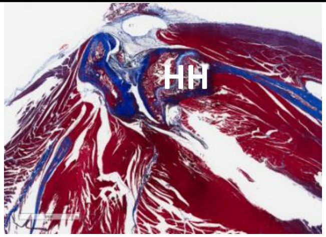	No significant pathology	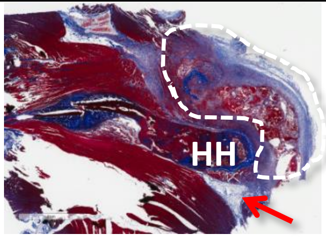	Extensive necrosis, hemorrhage, fibrosis and inflammatory cell infiltrate. Joint is not readily identified in the tissue section due to extensive tissue injury.

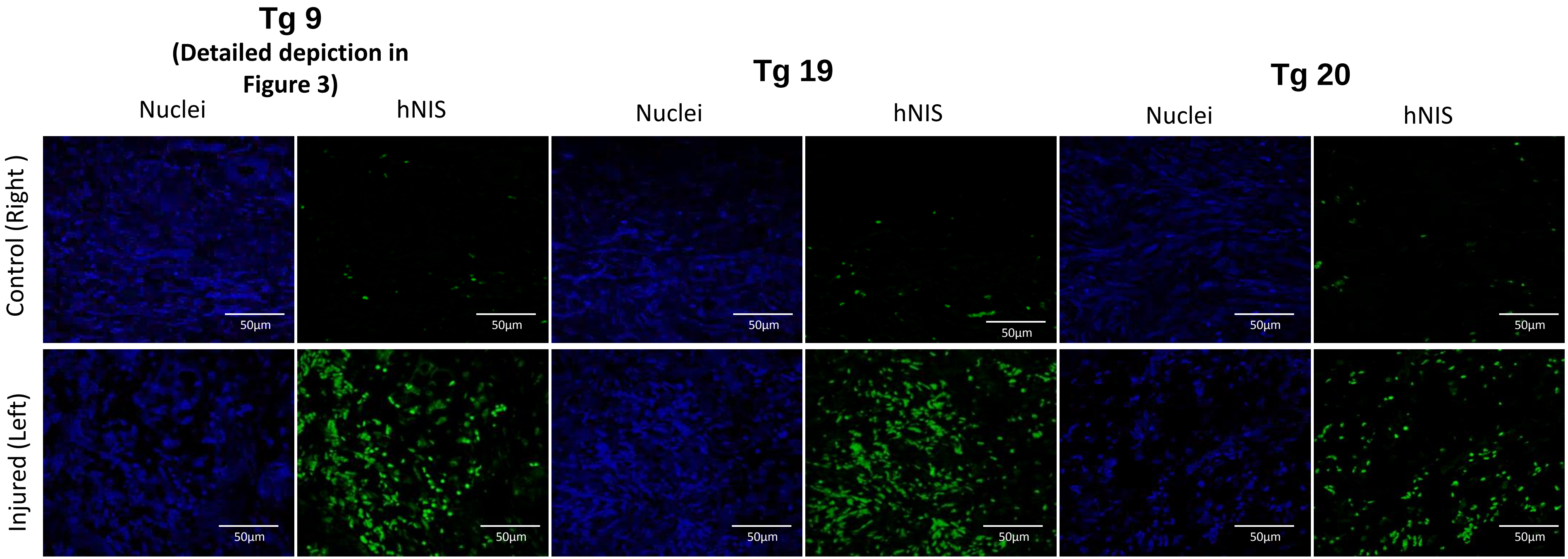

Supplementary Figure 2. The rotator cuff Injured shoulder of the Col1α1-hNIS transgenic rats has increased hNIS immunofluorescent staining. Confocal images were taken at 40X magnification following staining paraffin embedded rotator cuff tissue sections with Hoeschst stain for nuclei (Blue) or an affinity purified polyclonal anti-hNIS antibody (Green) from three individual Col1α1-hNIS transgenic rats.

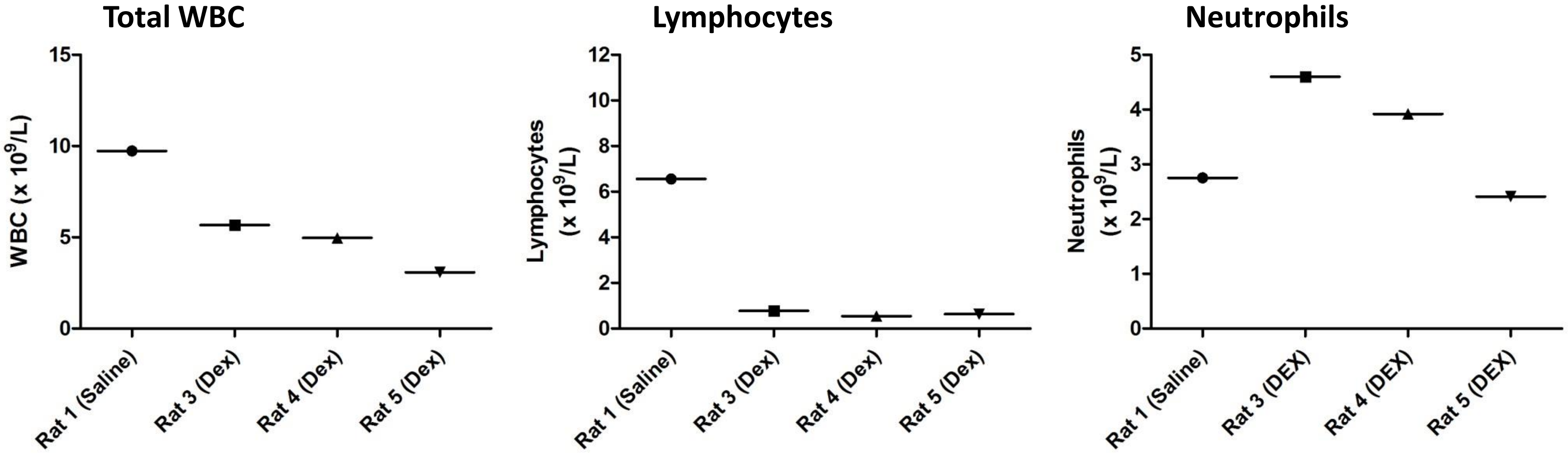

Supplementary Figure 3. Dexamethsone treatment causes immunosuppression in Sprague Dawley treated rats as measured by total blood cell counts. Peripheral blood was collected 5 days following initial dexamethasone (2 mg/kg) or saline treated rats. Complete blood count (CBC) analysis was done to determine total white blood cell counts, lymphocytes and neutrophils presence in the peripheral blood samples.
